# Data supporting the reconstruction study of missing wind speed logs using wavelet techniques for getting maximum likelihood

**DOI:** 10.1016/j.dib.2020.105835

**Published:** 2020-06-11

**Authors:** Dora Cama-Pinto, Pastor David Chávez-Muñoz, Andres Felipe Solano-Escorcia, Alejandro Cama-Pinto

**Affiliations:** a Department of Computer Architecture and Technology, University of Granada, 18071 Granada, Spain; b Department of Engineering, Pontificia Universidad Católica del Perú, Av. Universitaria 1801, San Miguel, Lima 32, Lima, Perú; c Department of Computer Science and Engineering Electronic, Universidad de la Costa, Calle 58 No. 55-66, Barranquilla, Colombia

**Keywords:** Wind data, Wavelet transform, Fast Fourier transform, Missing data, Renewable energy, Data filling

## Abstract

The data to construct the missing wind-speed value in the weather station record at “Collado de Yuste”, between the years 2002 to 2012, was calculated using wind speed data recorded in two other nearby weather stations, those in “Solana del Zapatero” and “Calar Alto”. The three mentioned stations are located in the mountain range of the province of Almeria, Autonomous Community of Andalusia, Spain. After calculating the degree of association using the correlation coefficient [1] and Wavelet Transform Scalogram [2], the data was successfully constructed. This paper refers to another study: Wind missing data arrangement using wavelet based techniques for getting maximum likelihood [3].

Specifications table

**Table:** 

**Subject**	Renewable Energy, Sustainability and the Environment Energy Engineering and Power Technology
**Specific subject area**	Wind Power, Wavelet Transform to analyze wind speed records
**Type of data**	Table Images
**How data were acquired**	The average wind speed data were acquired from three weather stations located at different points in the Sierra de Almeria, Andalusia, Spain.
**Data format**	Raw Calculated
**Parameters for data collection**	Average wind speed data was recorded using the anemometer at three weather stations.
**Description of data collection**	In three different meteorological stations in the highlands of Almeria, were recorded with its respective anemometers, the wind speed every 600 s (10 min). “Calar Alto” data was stored between January 1st, 2002 and December 31st, 2009. At “Solana del Zapatero” and “Collado de Yuste” stations data was stored between January 1st, 2002 and December 31st, 2012. All these records are in Original_Values.xls. The reconstructed record of "Collado de Yuste" is in the file Reconstructed_Values.xls. Both located in http://data.mendeley.com.
**Data source location**	Country: Almeria/SpainThe data were obtained from weather stations located in the province of Almeria, called 1) “Collado de Yuste” (37.22633 N, 2.430768 W, 1866 masl) referred as CY 2) “Solana del Zapatero” (37.31286 N, 2.430768 W, 1116.1 masl) referred as SZ 3) “Calar Alto” (37.22099 N,−2.548748 W, 2151 masl) referred as CA
**Data accessibility**	Direct URL to data: Mendeley Data, http://data.mendeley.com/datasets/v3kzgr9rxb/2
**Related research article**	Author's name: Dora Cama-Pinto, Antonio J. Zapata-Sierra, Francisco Manzano Agugliaro, Alejandro Cama-Pinto Title: Wind missing data arrangement using wavelet based techniques for getting maximum likelihood. Journal: Energy Conversion and Management. https://doi.org/10.1016/j.enconman.2019.01.109

## Value of the Data

• This data can be used to understand how to reconstruct missing data in wind speed records verifying their naturalness with respect to the original values.
• This data contains essential detail concerning the non-stationary behavior of wind. Therefore, this research will contribute towards an improvement in our understanding of the wind energy field, which by 2023 will represent 12.4% of renewable energies [4].
• This data is a useful contribution to the prediction of wind behaviour and wind energy potential.
• This data can be used for the preliminary assessment of wind farm projects' viability.

## 1 Data

**Fig. 1:**
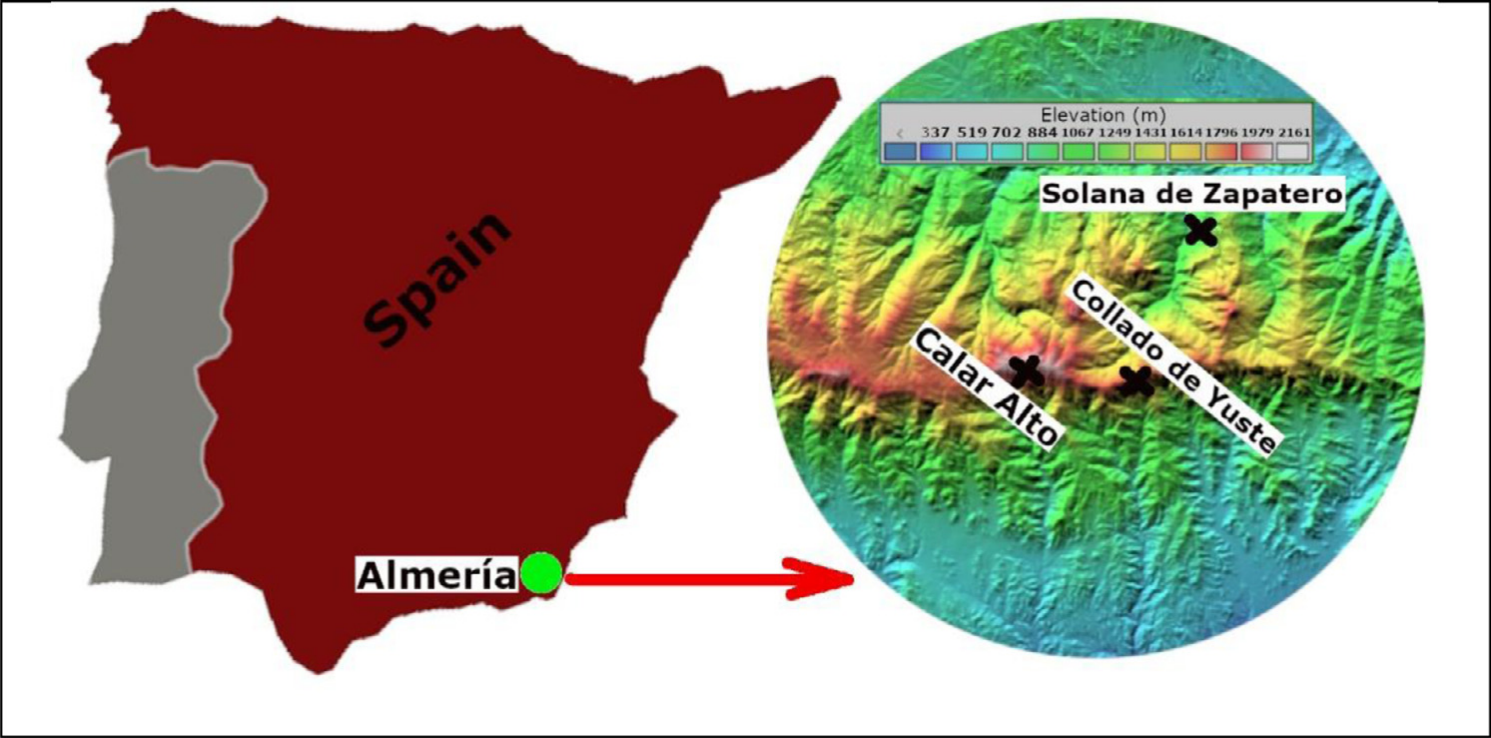
Locations of Collado de Yuste, Solana del Zapatero and Calar Alto weather stations in Almeria.

The data set is stored on Mendeley's data website (https://data.mendeley.com) and is organized in two Excel sheet files: Original_Values.xls (file with average wind speed record in the three locations without applying any recovery technique) and reconstructed_wind.xls (file with reconstructed average wind speed record in the location of “Collado de Yuste”). The information set in Original_Values.xls contains data records of the average wind speed collected by the weather stations of “Calar Alto” at 2151 masl (located in the tab “Calar Alto”), “Solana del Zapatero” at 1116.1 masl (located in the tab “Solana del Zapatero”) and “Collado de Yuste” at 1866 masl (located in the tab “Collado de Yuste”) in the Sierra de Almeria, Andalusia, Spain. Shown in Fig. 1.

Average wind speed data from “Calar Alto” weather station are from the years 2002–2009. “Solana del Zapatero” and “Collado de Yuste” records are both from years 2002–2012.

To fill in missing CY data, we employ interpolation using data from nearby stations. For this purpose, is used the equation 1:

(1) $$K_{i}=\frac{1}{M}\sum_{i=1}^{i=M}\alpha_{i}C_{i}$$

The meaning of each element of Eq. (1) is explained in Table 1.

**Table 1:** Explanation for Eq. (1) elements.

Variable	Explanation
K*i*	Lost data to reconstruct it in a specific date and time for CY
*Ci*	Reference data from a nearby station i in the same specific date and time of Ki
M	Number of locations with existing data for a specific date and time.
*αi*	Weighting coefficient. It is the annual value rate of the CY median in relation to one of the nearby stations

The initial average hourly values during each of the eleven years in Table 2 and in the 12 months of the 11 years of measurement in Table 3. After reconstructing the missing values in the CY register, Tables 3 and 4 are modified with the new values shown in Tables 4 and 5 respectively. The scalogram generated with the original CY record and with the reconstructed CY values, the FFT (Fast Fourier Transform) of the original CY information and the FFT including the reconstructed values, giving us a significant graphic contrast, we can see it graphically in [3].

**Table 2:** Average wind speed values (m/s) recorded at the CY location from 0 to 24 h.

Hour	2002.	2003	2004	2005	2006	2007	2008	2009	2010	2011	2012
00–01	4.6	3.8	4.6	4.2	4.5	4.3	4.2	1.9	3.5	4.2	4.5
01–02	4.7	3.9	4.5	4.2	4.4	4.2	4.1	1.8	3.4	4.2	4.2
02–03	4.8	3.8	4.5	4.1	4.4	4.2	4.1	1.8	3.4	4.1	4.4
03–04	4.7	3.7	4.3	4.2	4.4	4.2	4.0	1.8	3.5	4.0	4.4
04–05	4.6	3.6	4.4	4.1	4.4	4.0	3.9	1.8	3.5	4.0	4.4
05–06	4.6	3.6	4.6	4.0	4.4	3.9	3.9	1.9	3.5	4.0	4.4
06–07	4.6	3.6	4.6	4.0	4.3	4.1	4.0	1.9	3.7	4.0	4.5
07–08	4.7	3.6	4.6	3.9	4.3	4.2	4.0	1.9	3.7	4.0	4.5
08–09	4.6	3.7	4.5	4.1	4.3	4.2	4.0	1.9	4.0	4.0	4.5
09–10	4.6	3.8	4.6	4.1	4.4	4.2	4.0	1.9	4.5	4.1	4.4
10–11	4.6	3.8	4.6	4.2	4.3	4.1	4.0	1.8	4.8	4.0	4.0
11–12	4.5	3.9	4.6	4.1	4.2	4.1	3.9	1.8	4.8	4.0	4.4
12–13	4.6	3.9	4.5	4.1	4.2	4.0	3.9	1.7	4.8	4.0	4.5
13–14	4.6	3.8	4.3	4.2	4.3	3.9	3.8	1.7	4.8	4.0	4.3
14–15	4.5	3.8	4.3	4.1	4.2	3.9	3.8	1.7	4.8	3.8	4.3
15–16	4.5	3.8	4.3	4.2	4.1	3.9	3.8	1.7	4.7	3.8	4.2
16–17	4.4	3.8	4.5	4.1	4.2	4.0	3.9	1.7	4.7	3.8	4.2
17–18	4.4	3.7	4.5	4.2	4.2	4.0	4.0	1.8	4.6	3.9	4.3
18–19	4.5	3.7	4.5	4.3	4.3	4.0	4.0	1.8	4.4	3.9	4.4
19–20	4.4	3.8	4.6	4.3	4.4	4.2	4.1	1.8	4.2	4.0	4.6
20–21	4.5	3.9	4.6	4.4	4.4	4.3	4.1	1.8	4.0	4.1	4.6
21–22	4.6	3.9	4.5	4.3	4.4	4.2	4.2	1.8	3.8	4.2	4.6
22–23	4.6	3.9	4.5	4.3	4.5	4.3	4.2	1.8	3.6	4.3	4.5
23–24	4.7	3.9	4.5	4.2	4.5	4.3	4.3	1.7	3.6	4.3	4.6

**Table 3:** Average wind speed values (m/s) per month in CY location from 0 to 24 h.

Hour	Jan.	Feb.	Mar.	Apr.	May.	Jun.	Jul.	Aug.	Sep.	Oct.	Nov.	Dec.
00–01	4.2	3.8	4.3	5.1	5.1	4.6	3.1	3.7	4.8	5.3	6.3	5.5
01–02	4.7	3.9	4.1	5.1	5.4	4.4	2.9	3.7	4.9	5.3	6.5	5.2
02–03	4.8	4.3	3.9	5.4	5.5	4.9	2.8	3.6	4.8	4.9	6.7	5.7
03–04	4.8	4.4	3.8	5.1	5.2	5.1	2.8	3.3	4.8	4.8	6.3	5.5
04–05	4.7	4.2	3.8	5.3	5.4	5.2	2.9	2.9	4.7	5.1	6.2	5.1
05–06	4.4	4.4	3.7	5.5	5.2	5.4	2.9	2.9	4.8	4.8	6.6	5.0
06–07	4.2	4.1	3.7	5.1	4.9	5.1	2.9	3.3	4.5	4.9	7.0	5.5
07–08	4.3	4.1	3.9	4.9	4.6	4.7	3.2	3.1	4.6	5.0	7.6	6.2
08–09	4.1	4.3	3.8	4.6	4.7	4.4	3.0	3.1	4.2	5.0	7.6	6.6
09–10	3.8	4.4	3.7	4.7	5.2	4.3	2.8	3.1	4.5	5.4	7.0	6.7
10–11	3.8	3.4	3.6	4.8	5.6	4.6	2.6	3.0	4.6	5.5	7.2	6.9
11–12	3.7	3.3	3.7	4.6	5.6	4.8	2.2	2.9	4.2	5.5	6.7	6.6
12–13	3.4	3.5	3.7	4.6	5.7	5.0	2.2	2.8	4.1	5.7	7.1	6.9
13–14	3.2	3.5	4.0	4.8	5.3	4.8	2.6	2.8	4.0	6.0	7.0	7.2
14–15	3.2	3.4	3.9	4.6	4.8	4.4	2.4	3.1	3.9	5.8	7.2	7.8
15–16	3.2	3.1	3.8	4.7	4.4	4.2	2.2	2.9	4.2	5.7	7.2	7.8
16–17	2.9	3.6	4.0	4.9	4.4	3.8	2.4	2.8	4.4	5.5	7.3	7.2
17–18	3.0	3.9	4.1	5.1	4.4	4.0	2.4	2.8	4.6	5.1	6.9	6.7
18–19	3.0	4.1	4.0	5.2	4.4	4.5	2.4	2.8	4.6	5.5	6.6	6.6
19–20	3.4	4.2	3.6	5.3	4.9	4.7	2.3	2.8	4.3	5.8	5.9	6.2
20–21	3.7	4.1	3.9	5.2	5.4	4.9	2.5	2.9	3.9	5.6	6.2	6.1
21–22	3.7	4.1	4.0	5.4	5.3	5.2	2.9	3.0	4.2	5.2	6.8	5.8
22–23	3.5	4.1	4.0	5.0	5.5	5.3	3.1	3.1	4.3	4.9	6.2	5.9
23–24	3.7	3.9	4.4	5.0	5.4	5.2	3.0	3.6	4.5	5.1	6.3	5.7

**Table 4:** Average wind speed values (m/s) including reconstructed data in CY location from 0 to 24 h.

Hour	2002.	2003	2004	2005	2006	2007	2008	2009	2010	2011	2012
00–01	3.7	10.1	10.7	3.1	16.3	0.7	4.6	2.1	15.0	3.1	1.9
01–02	2.0	11.5	8.8	4.0	13.5	0.0	4.6	1.7	14.8	3.4	3.4
02–03	1.2	12.0	7.2	3.7	10.8	0.0	4.9	1.7	13.4	2.4	1.1
03–04	2.1	11.3	5.3	3.5	9.1	0.0	5.2	1.0	11.9	3.2	1.1
04–05	2.3	9.4	4.7	4.3	10.3	0.0	5.2	1.5	12.5	4.5	0.9
05–06	3.6	7.6	4.9	5.0	10.4	1.4	3.9	2.9	11.9	3.2	0.8
06–07	4.0	8.4	5.6	4.1	9.4	3.2	4.1	3.8	11.5	3.1	0.1
07–08	4.4	8.3	5.7	3.5	10.8	3.3	2.3	3.9	16.0	6.7	1.2
08–09	5.2	7.7	5.3	2.8	11.4	1.9	2.0	3.6	24.3	3.9	2.0
09–10	5.4	7.5	3.8	3.3	12.7	1.3	2.7	4.8	25.3	2.1	2.7
10–11	4.6	6.5	4.0	4.0	9.1	1.2	2.8	3.8	25.3	2.9	2.9
11–12	4.6	7.7	4.6	4.2	9.6	3.7	2.3	5.6	22.8	4.5	2.2
12–13	4.3	6.8	6.0	4.2	11.9	3.2	1.4	5.7	20.9	5.1	2.5
13–14	5.1	5.6	6.1	3.7	7.2	2.9	0.6	5.4	20.5	4.1	2.3
14–15	4.6	2.8	4.4	2.8	10.1	2.7	1.7	6.1	17.5	4.5	1.2
15–16	5.3	5.3	6.9	5.0	8.7	2.5	1.5	5.6	15.9	4.8	1.2
16–17	5.6	3.1	8.3	6.3	7.6	3.0	2.6	6.2	11.6	5.2	1.8
17–18	5.2	3.2	7.7	6.2	7.7	5.1	4.6	4.9	9.3	6.0	1.1
18–19	5.1	1.8	6.4	6.0	9.4	3.7	3.6	3.1	10.2	6.8	3.0
19–20	6.0	1.2	5.0	4.9	8.1	5.5	4.1	3.1	8.2	6.6	3.6
20–21	6.6	2.2	7.5	4.2	7.7	4.0	1.5	3.7	13.0	5.3	4.1
21–22	6.1	3.6	6.3	4.2	9.4	4.3	1.1	4.4	15.4	4.6	3.5
22–23	6.7	4.5	4.4	4.6	10.5	3.0	3.8	3.8	8.3	4.3	4.7
23–24	6.6	2.6	5.3	4.6	10.5	1.6	4.6	4.7	7.8	4.8	5.5

**Table 5:** Average wind speed values (m/s) per month including reconstructed data in CY location from 0 to 24 h.

Hour	Jan.	Feb.	Mar.	Apr.	May.	Jun.	Jul.	Aug.	Sep.	Oct.	Nov.	Dec.
00–01	4.2	4.0	4.9	5.1	5.1	4.6	4.2	4.6	4.8	5.3	6.3	5.5
01–02	4.7	4.2	4.5	5.1	5.4	4.4	4.1	4.5	4.9	5.3	6.5	5.2
02–03	4.8	4.5	4.2	5.4	5.5	4.9	3.9	4.3	4.8	4.9	6.7	5.7
03–04	4.8	4.7	4.0	5.1	5.2	5.1	3.8	4.0	4.8	4.8	6.3	5.5
04–05	4.7	4.6	4.1	5.3	5.4	5.2	3.5	3.5	4.7	5.1	6.2	5.1
05–06	4.4	4.7	4.1	5.5	5.2	5.4	3.7	3.5	4.8	4.8	6.6	5.0
06–07	4.2	4.4	4.1	5.1	4.9	5.1	3.7	3.9	4.5	4.9	7.0	5.5
07–08	4.3	4.5	4.3	4.9	4.6	4.7	3.9	3.8	4.6	5.0	7.6	6.2
08–09	4.1	4.6	4.2	4.6	4.7	4.4	3.8	3.7	4.2	5.0	7.6	6.6
09–10	3.8	4.9	4.1	4.7	5.2	4.3	3.7	3.6	4.5	5.4	7.0	6.7
10–11	3.8	3.8	4.1	4.8	5.6	4.6	3.5	3.6	4.6	5.5	7.2	6.9
11–12	3.7	3.7	4.1	4.6	5.6	4.8	3.0	3.6	4.2	5.5	6.7	6.6
12–13	3.4	3.8	4.2	4.6	5.7	5.0	3.1	3.4	4.1	5.7	7.1	6.9
13–14	3.2	3.9	4.5	4.8	5.3	4.8	3.4	3.6	4.0	6.0	7.0	7.2
14–15	3.2	3.9	4.4	4.6	4.8	4.4	3.2	3.8	3.9	5.8	7.2	7.8
15–16	3.2	3.7	4.2	4.7	4.4	4.2	3.0	3.5	4.2	5.7	7.2	7.8
16–17	2.9	4.3	4.5	4.9	4.4	3.8	3.2	3.4	4.4	5.5	7.3	7.2
17–18	3.0	4.5	4.6	5.1	4.4	4.0	3.4	3.5	4.6	5.1	6.9	6.7
18–19	3.0	4.7	4.4	5.2	4.4	4.5	3.5	3.6	4.6	5.5	6.6	6.6
19–20	3.4	4.8	3.9	5.3	4.9	4.7	3.5	3.6	4.3	5.8	5.9	6.2
20–21	3.7	4.5	4.2	5.2	5.4	4.9	3.8	3.8	3.9	5.6	6.2	6.1
21–22	3.7	4.4	4.3	5.4	5.3	5.2	4.2	3.8	4.2	5.2	6.8	5.8
22–23	3.5	4.5	4.2	5.0	5.5	5.3	4.4	4.0	4.3	4.9	6.2	5.9
23–24	3.7	4.2	4.7	5.0	5.4	5.2	4.4	4.5	4.5	5.1	6.3	5.7

## 2 Experimental design, materials, and methods

The initial record of the "Collado de Yuste" weather station shows 62.108 lost data in wind speed average measurement. Through mathematical calculation based on interpolating data from the other two nearby stations, explained in [3] the record was reconstructed, lowering the loss rate to 447 data. Raw records from the three weather stations show missing data. The method used to reconstruct missing wind speed data from nearby records from other stations could contribute to improve the study for wind generation in Spain and anywhere in the world.

The methodology applied to reconstruct the missing wind speed data in the “Collado del Yuste” station record is:

1. Download the files in Excel format with the initial records of the average wind speeds of the three weather stations (“Calar Alto”, “Solana del Zapatero” and “Collado de Yuste”) called “Original_Values.xls”.
2. Reading of variables and extraction: The characteristics of the downloaded Excel files, the structure of the matrix (records every 10 min from 2002 to 2012) require advanced software tools. In this study, the Matlab software [5] was used to read and process the table contents.
3. Calculation: Reconstructing the average wind speed record at “Collado de Yuste” station (“Reconstructed_Values.xls”) from the values stored at “Calar Alto” and “Solana del Zapatero” weather stations were calculated using the equations related in the investigation [3]. This reconstructed record shows hourly average values during each of the eleven years in Table 3 and in the 12 months of the 11 years of measurement in Table 4.
4. Validation: The naturalness of the reconstructed data is validated through the power spectral density (PSD) provided by the Fast Fourier Transform (FFT) and the Wavelet transform scalogram (see in [3]).

## Declaration of Competing Interest

The authors declare that they have no known competing financial interests or personal relationships which have, or could be perceived to have, influenced the work reported in this article.

## Appendix Supplementary materials

Image, application 1
